# Effect of Self-Assembly of Fullerene Nano-Particles on Lipid Membrane

**DOI:** 10.1371/journal.pone.0077436

**Published:** 2013-10-29

**Authors:** Saiqun Zhang, Yuguang Mu, John Z. H. Zhang, Weixin Xu

**Affiliations:** 1 Institutes for Advanced Interdisciplinary Research, East China Normal University, Shanghai, China; 2 School of Biological Sciences, Nanyang Technological University, Singapore; 3 State Key Laboratory of Precision Spectroscopy, East China Normal University, Shanghai, China; 4 Department of Chemistry, New York University, New York, New York, United States of America; Brandeis University, United States of America

## Abstract

Carbon nanoparticles can penetrate the cell membrane and cause cytotoxicity. The diffusion feature and translocation free energy of fullerene through lipid membranes is well reported. However, the knowledge on self-assembly of fullerenes and resulting effects on lipid membrane is poorly addressed. In this work, the self-assembly of fullerene nanoparticles and the resulting influence on the dioleoylphosphtidylcholine (DOPC) model membrane were studied by using all-atom molecular dynamics simulations with explicit solvents. Our simulation results confirm that gathered small fullerene cluster can invade lipid membrane. Simulations show two pathways: 1) assembly process is completely finished before penetration; 2) assembly process coincides with penetration. Simulation results also demonstrate that in the membrane interior, fullerene clusters tend to stay at the position which is 1.0 nm away from the membrane center. In addition, the diverse microscopic stacking mode (i.e., equilateral triangle, tetrahedral pentahedral, trigonal bipyramid and octahedron) of these small fullerene clusters are well characterized. Thus our simulations provide a detailed high-resolution characterization of the microscopic structures of the small fullerene clusters. Further, we found the gathered small fullerene clusters have significant adverse disturbances to the local structure of the membrane, but no great influence on the global integrity of the lipid membrane, which suggests the prerequisite of high-content fullerene for cytotoxicity.

## Introduction

Over the past decades, carbon nanoparticles’ applications in industrial and pharmaceutical industries have been developed rapidly. [1]–[6] Carbon nanoparticles, especially fullerenes and carbon nanotubes, which have special characteristic, mechanical and electric properties, are extensively studied in the scientific and industrial field. [7]–[9] These materials show a number of interesting properties such as the solubility in numerous organic solvents, [10] mechanical and electrical conductivity. [11] In aqueous system, fullerene is neither like a single molecule, nor like the bulk solid. Its behavior deviates from the similar hydrophobic substance (hydrophobic polyaromatic hydrocarbons). [12] Consequently, both in the nano-materials science and nano-drugs, carbon nanoparticles have broad applications. Specifically, with appropriate modifications, fullerene and its derivates can be utilized as gene delivery reagents, [13] HIV protease inhibitors, [14] high efficiency magnetic resonance imaging contrast agents, [15] animal model of ischemic brain injury, and the degenerating dopaminergic neuron model of Parkinson’s disease. [16], [17].

Meanwhile, of equal importance, the effects of nanoparticles on biological and natural environment bring a hot concern of biosafety. It is reported that the fullerene molecules are detected in emissions from the coal-fired power plants, [18] and when these fullerene molecules penetrate into the groundwater system, the entire aquatic biological systems, the plants, as well as our drinking water are affected. Sayes *et al.* and Oberdörster have observed that pure fullerenes in an aggregate form, which are weakly soluble in water, are healthily and environmentally dangerous. [19]–[21] In particular, it is found that fullerene aggregates exhibit toxicity towards fish and human skin, and liver cells as well as lung tissue. [19] Fullerenes in water mutually attract to generate nanoscaled colloidal structure, which is essentially crystalline in order with a simple hexagonal unit. [12], [22]–[24] They may interact with biomolecules and enter living cells in a different way compared to single fullerene. [25]–[28] So far, the mechanism of the fullerene cytotoxicity is still unclear. Studies on the interaction of the nanoparticles with cell membrane may provide a key to understand the basic questions in nanotoxicology and nanopharmacology.

Therefore, to know the mechanisms on the self-assembly and translocation of fullerene nanoparticles through lipid membranes, as well as resulting influences is scientifically meaningful. Although there are many experimental studies of the cell membrane damage by fullerene, [26], [29]–[32] direct evidence on the location of fullerenes in real cell membranes is missing and the related detailed microscopic mechanisms are lacked. On the other hand, computer simulation study is able to provide intuitive results, by ignoring the complexity of cell membrane. [11], [25], [27], [28], [33]–[37] Previous studies have mainly focused on the following aspects: (1) How to improve the water solubility of fullerene and its derivatives by chemical modifications, and thus to reduce its toxicity; [27], [38], [39] (2) Permeation of monomeric fullerene into the lipid bilayer; [25], [26], [28], [34], [40] (3) Interactions between fullerene and water molecules, as well as the interactions among fullerenes. [26], [41]–[44] Nevertheless, the issues of carbon nano-aggregation kinetics and microscopic structure, the invading mechanism of fullerene cluster into the cell membrane, as well as the resulting influence of the aggregate on the lipid membrane are poorly addressed.

In the present study, we wish to address the above questions by the following three aspects: first, the assembly mechanism of fullerene clusters in the presence of a model membrane; second, the dynamic penetration process of fullerene clusters into the model membrane; third, the influences of the fullerene clusters on the structure and permeability of the model membrane. We utilize the DOPC lipid membrane and the fullerene as a model system to illustrate the idea. Our simulations show that the assembled fullerene clusters penetrate the lipid membrane by two manners. In the membrane interior, Fullerene clusters tend to stay at the position that is 1.0 nm away from the membrane center. The diverse microscopic assembly structures of small fullerene clusters are characterized. Further it is found that the invaded nano-cluster brings a large structural disturbance to the local region of the lipid membrane, while the global integrity of the lipid membrane is retained. Our simulation study reveals a microscopic picture on the self-assembly behaviors of fullerene nano-particles and resulting effect on the lipid membrane.

## Methods

### Simulation Setup

In this work, fullerene (C60 with radius 0.35 nm is used as the nano-particle. Based on the Amber force field, the carbon atoms of each fullerene molecule are modeled as neutral Lennard-Jones particles with a cross section as *σ*  = 0.34 nm and the depth of potential well as *ε*  = 0.36 kJ/mol. The DOPC lipid membrane is taken as the model cell membrane. The whole simulation system contains 128 DOPC lipids, 6 fullerenes and several thousands of water molecules. Initially, the fullerene particles and lipid membrane were spatially well separated, with the distances (defined as the distance between the center of mass of fullerene and the lipid membrane surface) of 2.0 nm. The 6 fullerenes were placed in the bulk water: three of which on the upper leaflet side and the other three on the lower leaflet side of the lipid membrane. The distance between 6 fullerenes is also far away (up to 2.0 nm) so that no clusters preexist. The combined systems were then solvated in a cubic periodic box. The TIP3P water model was used for solvation. The total numbers of atoms are 16641, 17664, and 360, for the cases of solvent, lipid membrane, and fullerene, respectively. In addition, a 100-ns simulation on a fully hydrated pure DOPC lipid system with 128 lipids (free of fullerene) was also performed as the reference for comparisons.

### Simulation Protocol

The method of molecular dynamics simulation has shown broad applications. [45]–[49] Here all molecular dynamics simulations were performed with the GROMACS package [50] using the all-atomic Amber03 force field. [51] All bonds involving hydrogen atoms were constrained in length according to the LINCS protocol. [52] This allowed the use of an integration step of 0.002 ps in simulations. Nonbonded pair lists were updated every 5 integration steps. Using a Berendsen thermostat, the bilayer lipids, fullerene carbon balls and water molecules were separately coupled to an external heat bath at 298 K with a relaxation time of 0.1 ps. The surface tension was maintained at 440 bar nm while the pressure perpendicular to the bilayer surface was kept at 1 bar using the Berendsen pressure coupling with a relaxation time of 1 ps and a water compressibility of 4.5×10^−5^ bar^−1^. The trajectories were output every 1 ps. Electrostatic interactions were treated with the particle mesh Ewald method with a cutoff of 0.9 nm, and a cutoff of 1.4 nm was used in the calculation of van der Waals interactions. Following energy minimization using the steepest descent, two 100-ps equilibrations were separately conducted under NVT ensemble and NPT ensemble to arrive at the correct temperature and reach the proper density. The subsequent production runs were all performed under NPT ensemble. The six simulations (labelled as S1 to S6) were started from the same initial structure using different seeding numbers for the initial velocities, and their total simulation time is 280, 327, 320, 280, 189 and 256 ns, respectively.

## Results

### Fullerene Aggregation Dynamics

**Figure 1:**
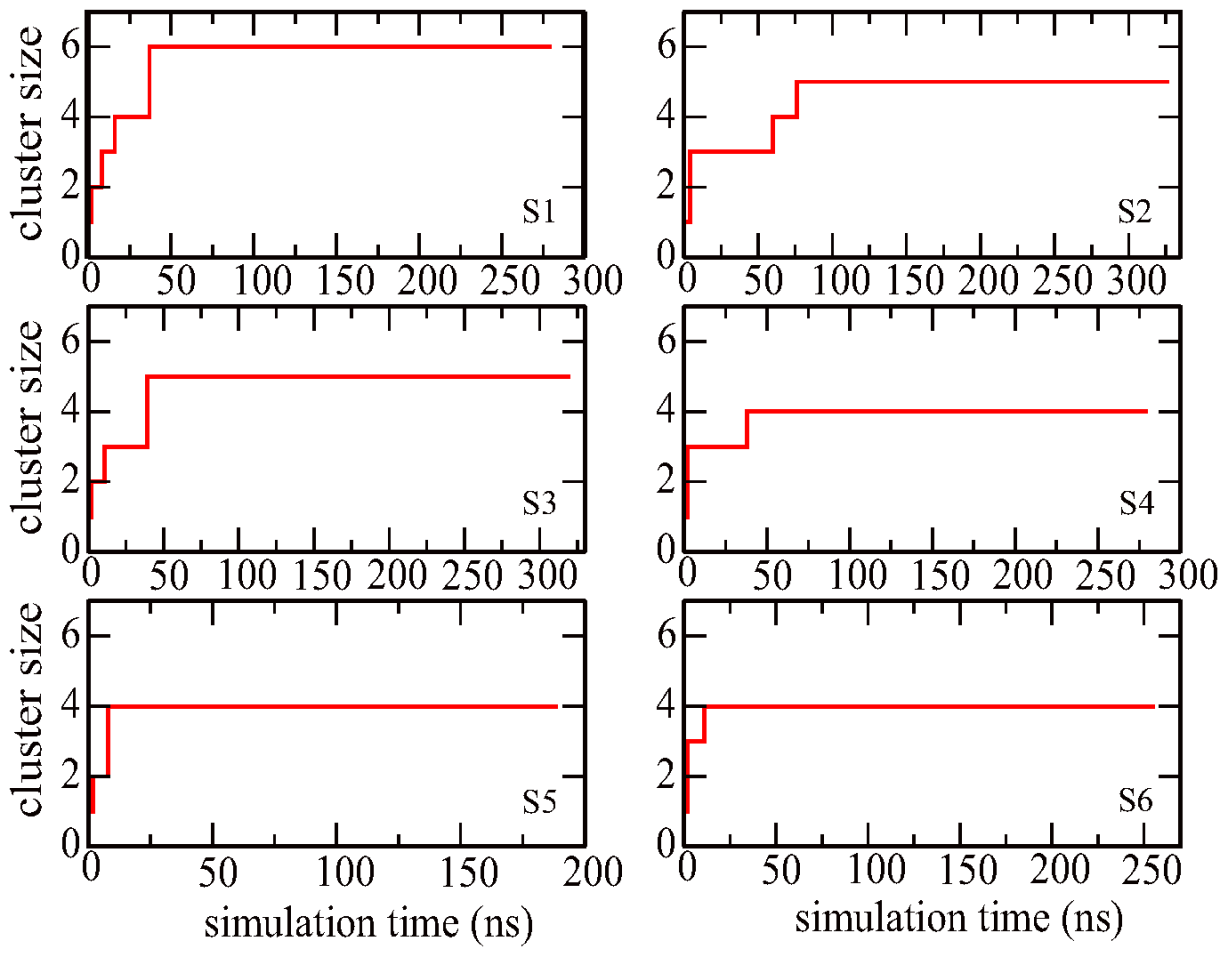
The largest cluster size as a function of simulation time. Y-axis is the amount of fullerene forming the largest cluster.

To investigate the self-assembly behavior of fullerenes, we calculated the time evolution of the largest cluster size of fullerene. The fullerenes were clustered by their spatial proximity, measured by the minimal distance between two fullerenes. The related distance cutoff was chosen to be 0.6 nm. The average largest cluster size as a function of simulation time is shown in Fig. 1. Each point on the curve is averaged over 1-ns simulation trajectory. The cluster sizes show a stepwise increase during the self-assembly process of fullerenes. In the early stage, the fullerenes quickly self-assembled into a small cluster because of the strong attractive interactions between fullerenes due to its hydrophobic property. The formed small cluster composed of 2∼3 particles acts as a nucleus/template for subsequent growth. It is however transient in water phase, whose lifetime is only about 5∼7 ns. Free fullerenes are subsequently added onto the nucleus to form a relatively bigger cluster. Once formed, the big cluster is stable and the cluster size does not change thereafter in all simulations. It is noticed that even in the penetrating process into lipid membrane, these big clusters do not dissociate, but remain the stable aggregation state. It is noticed that in one simulation trajectory (S1 in Fig. 1), all six fullerenes self-assembled into one cluster within 40 ns. Among other trajectories, after 100-ns simulations, two trajectories form a 5-fullerene aggregate while three trajectories form a 4-fullerene nano-cluster.

### Fullerene Stacking Patterns

**Figure 2:**
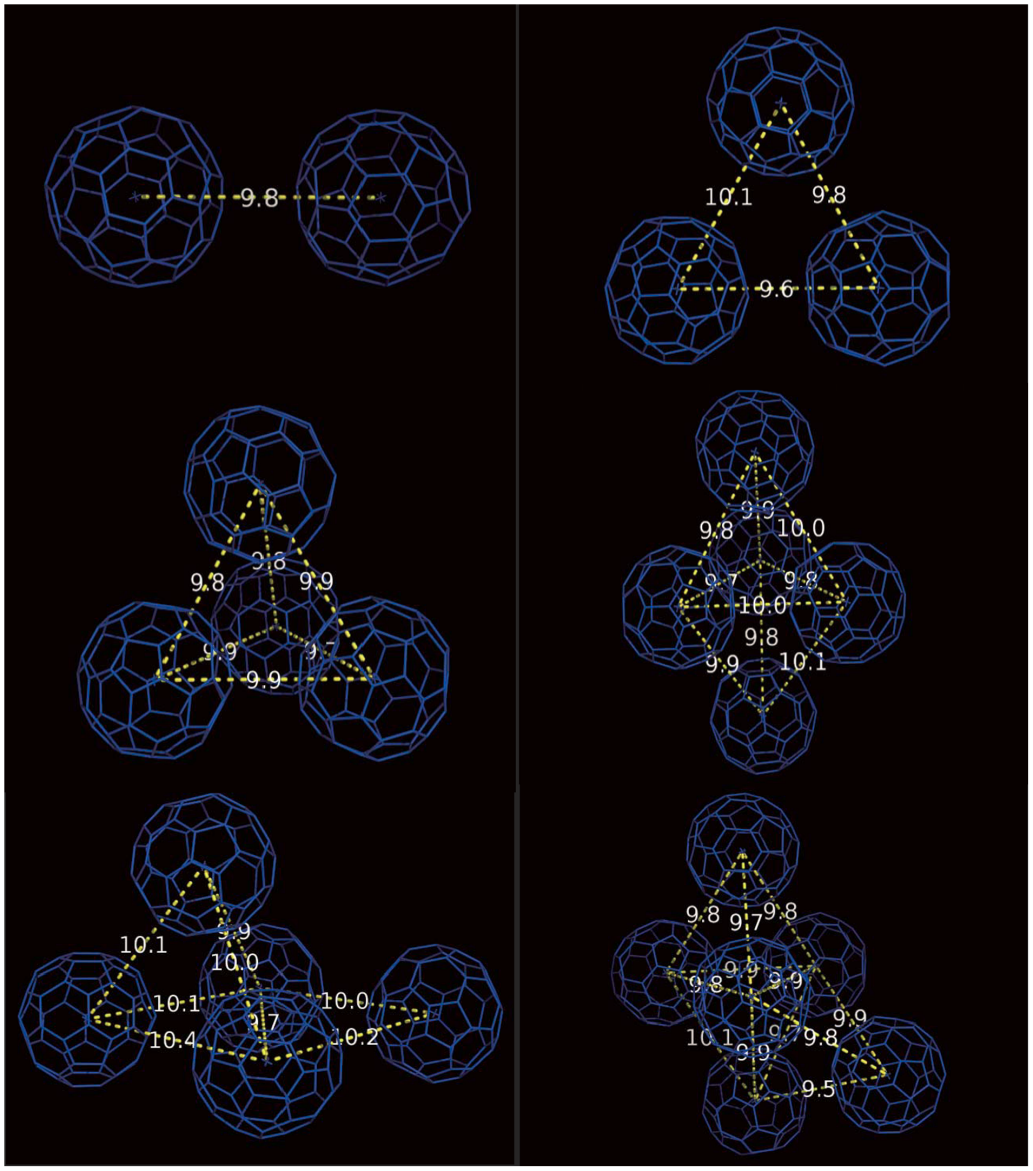
The equilibrated stacking mode of the fullerene aggregates. All the formed ordered assembly structures are listed here: the equilateral triangle (3 fullerenes); tetrahedron (4 fullerenes); deformed trigonal bipyramid and regular trigonal bipyramid (5 fullerenes) and deformed octahedron (6 fullerenes). The mutual distance is also shown.

Although the cluster size does not change after assembled, the cluster structure could be adjusted to obtain a stable stacking mode. From Fig. 2, one can see the equilibrated stacking patterns of the fullerene aggregates which have diverse three-dimensional structures. All the formed ordered assembly structures are listed here: the equilateral triangle (3 fullerenes); tetrahedron (4 fullerenes); deformed trigonal bipyramid and regular trigonal bipyramid (5 fullerenes) and deformed octahedron (6 fullerenes). Here deformation means that four of the five fullerenes form a regular tetrahedron and the fifth one interacts with the two underside particles to form a triangle. And thus this assembly is not in a regular trigonal bipyramid structure. Similarly, the so-called deformed octahedron consists of five fullerenes forming a regular trigonal bipyramid, and the sixth one with three of them constitute a tetrahedron. Thus, strictly speaking the octahedral structure is a combination of a tetrahedron and a triangular bipyramid. In summary, there are three kinds of equilibrated stacking modes: 6 fullerenes are aggregated into a deformed octahedral (S1); 5 fullerenes are aggregated into a deformed trigonal bipyramid (S2) and regular bipyramid (S3), 4 fullerenes are aggregated into a tetrahedral structure (S4–S6). As mentioned above, once formed, the fullerene cluster is stable and the cluster size does not change thereafter in all simulations. Even in the penetrating process, these stacking modes of fullerene aggregate are found well maintained. Unfortunately, our MD simulation cannot determine which aggregation state is more preferred to interact with lipid membrane. However, it is clear that large fullerene aggregates formed in an aqueous solution might not enter lipid membranes, while smaller ones are much easier to enter. [33], [53], [54].

Experimentally, Andrievsky and co-workers found that the fullerene clusters in the aqueous solution have the crystalline feature, among which the spherical 13-fullerene cluster has a crystalline phase with an fcc lattice. [55] By using electron diffraction, Fortner *et al.* also found that the fullerene aggregates are crystalline: small aggregates are usually circular in cross section; intermediate and large aggregates are rectangular; and the extremely large aggregates often appear to be triangular. [12] These results have also been confirmed by other studies. [56]–[59] In our simulation study, results based on a system with six fullerenes cannot be directly compared with experimental works. However, the crystalline nature of the fullerene cluster is also reflected in the simulated small fullerene cluster. And the experimental observations support the equilibrated stacking modes of fullerenes found in our simulations. In addition, theoretical studies of the fullerene cluster confirmed that the stable structures of fullerene aggregates have symmetry elements to decease the strain energy. [60]–[62] Our simulations, on the other hand, provide a detailed high-resolution characterization of the microscopic structures of the small fullerene clusters. Interestingly, Doye *et al.* investigated the putative global minima for (C60)*_N_* (N = 3∼105) clusters modelled by the Pacheco and Prates-Ramalho (PPR) potential, which is an improved single-site potential to describe many properties of bulk fullerene. [62] Their theoretical results showed that the global minima for (C60)*_N_* (N = 3∼6) was the equilateral triangle (3 fullerenes); tetrahedron (4 fullerenes); regular trigonal bipyramid (5 fullerenes) and octahedron (6 fullerenes) respectively, which well supports our MD simulation results.

Formation of the ordered assembly by fullerenes is determined by the balance of all physical interactions, leading to the thermodynamically stable fullerene cluster in both the water and lipid membrane phases. Specifically, formation of the equilibrium structure is mainly due to the lower strain energy and the preferred entropy. By using the intermolecular potential and carbon-carbon potential, Cirifalco *et al.* found that the equilibrium distance between two fullerene molecules in vacuum is about 1.0 nm, at which the energy is a minimum. [63] In our simulation study, the simulated equilibrium distance between two fullerene molecules in the small cluster is also close to 1.0 nm (Fig. 2). The deformation of the trigonal bipyramid and octahedron is likely due to the disturbance induced by the environment such as the water molecules and the lipids. Thus some distances are slightly deviated from the equilibrium value in vacuum.

### Penetration of Fullerenes into the DOPC Membrane

**Figure 3:**
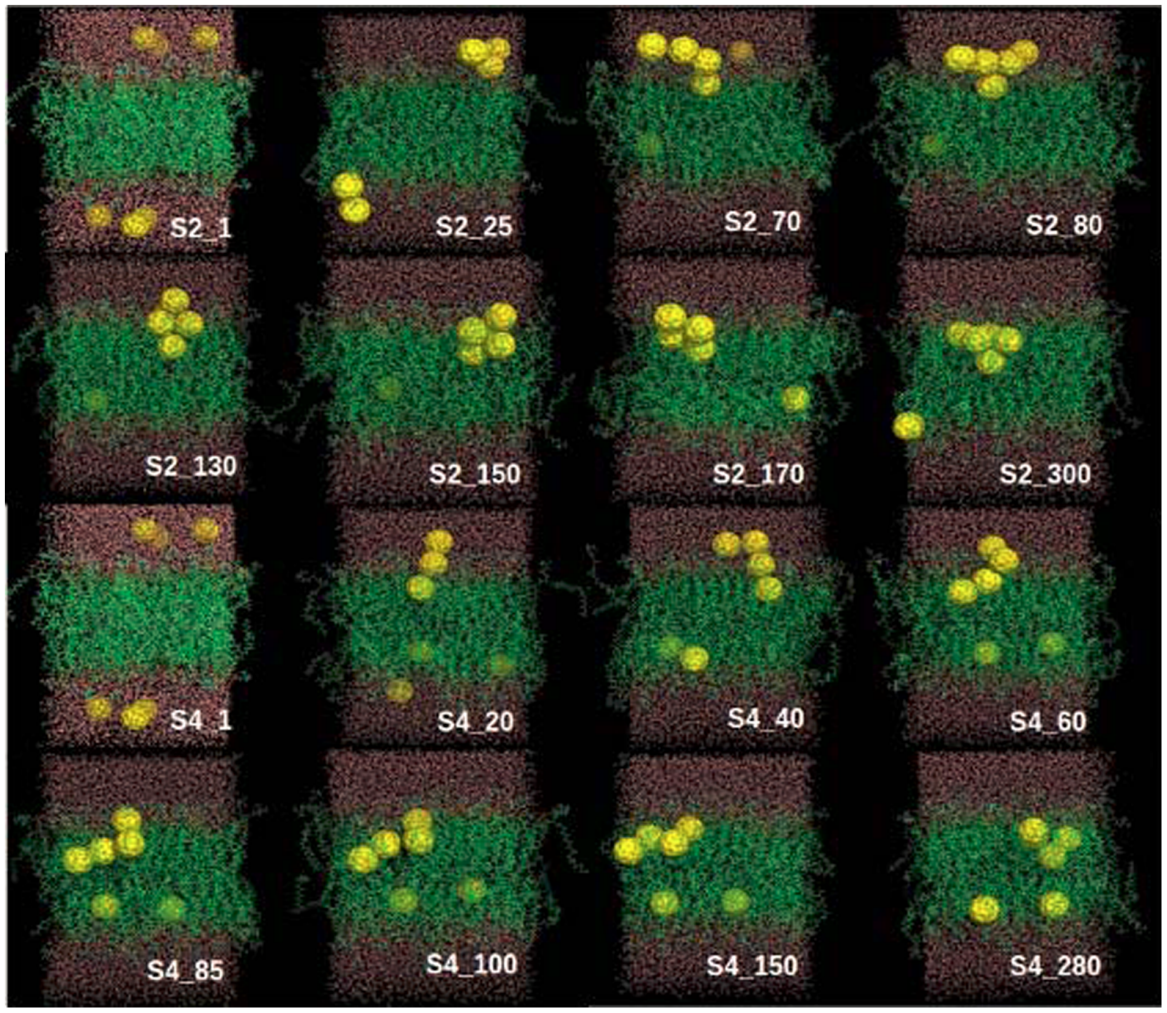
Eight representative snapshots selected according to the simulation time series (S2 and S4). The six fullerenes, DOPC lipids and water molecules are shown as yellow spheres, green lines and red lines, respectively.

**Figure 4:**
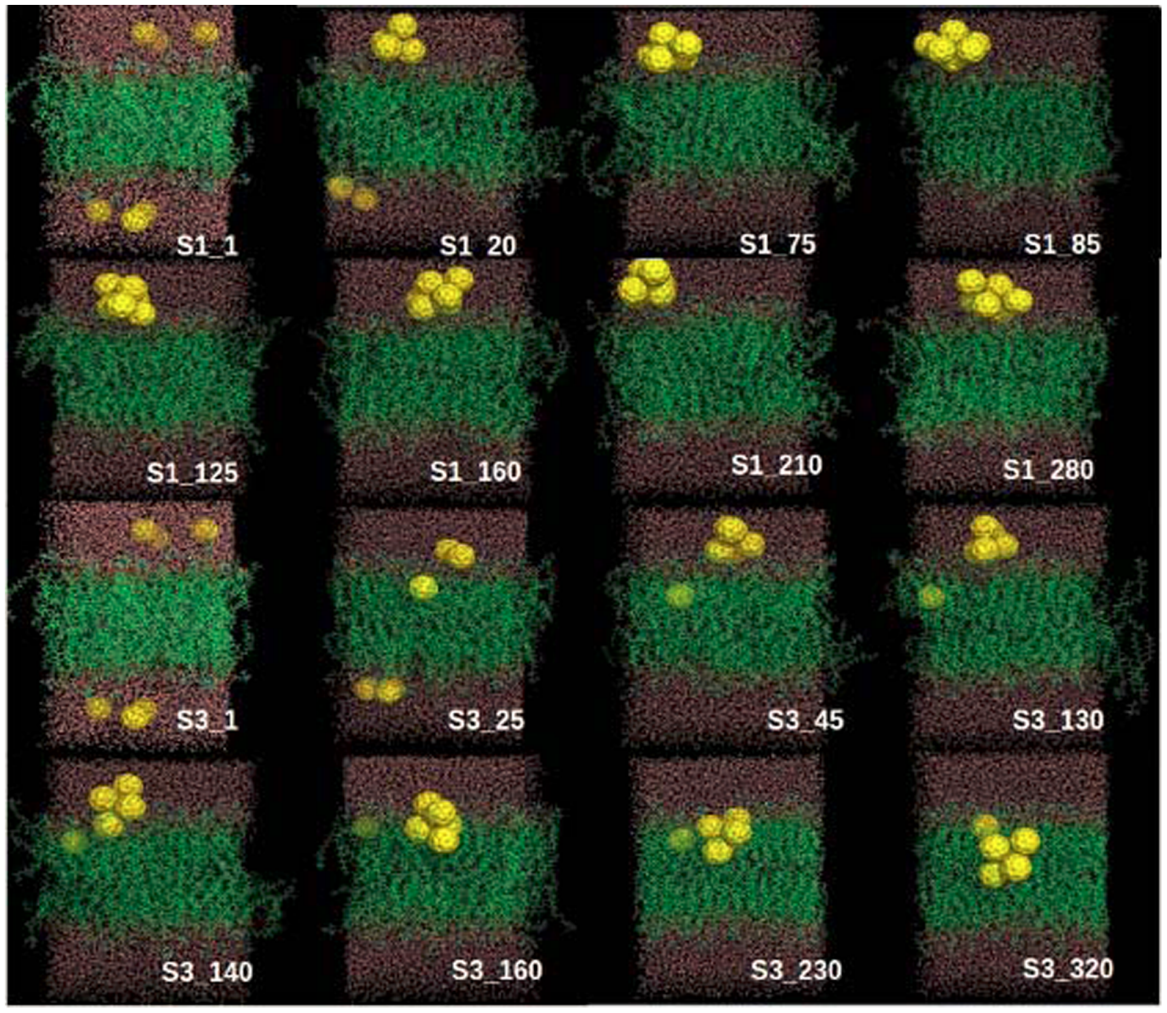
Eight representative snapshots selected according to the simulation time series (S1 and S3). The six fullerenes, DOPC lipids and water molecules are shown as yellow spheres, green lines and red lines, respectively.

**Figure 5:**
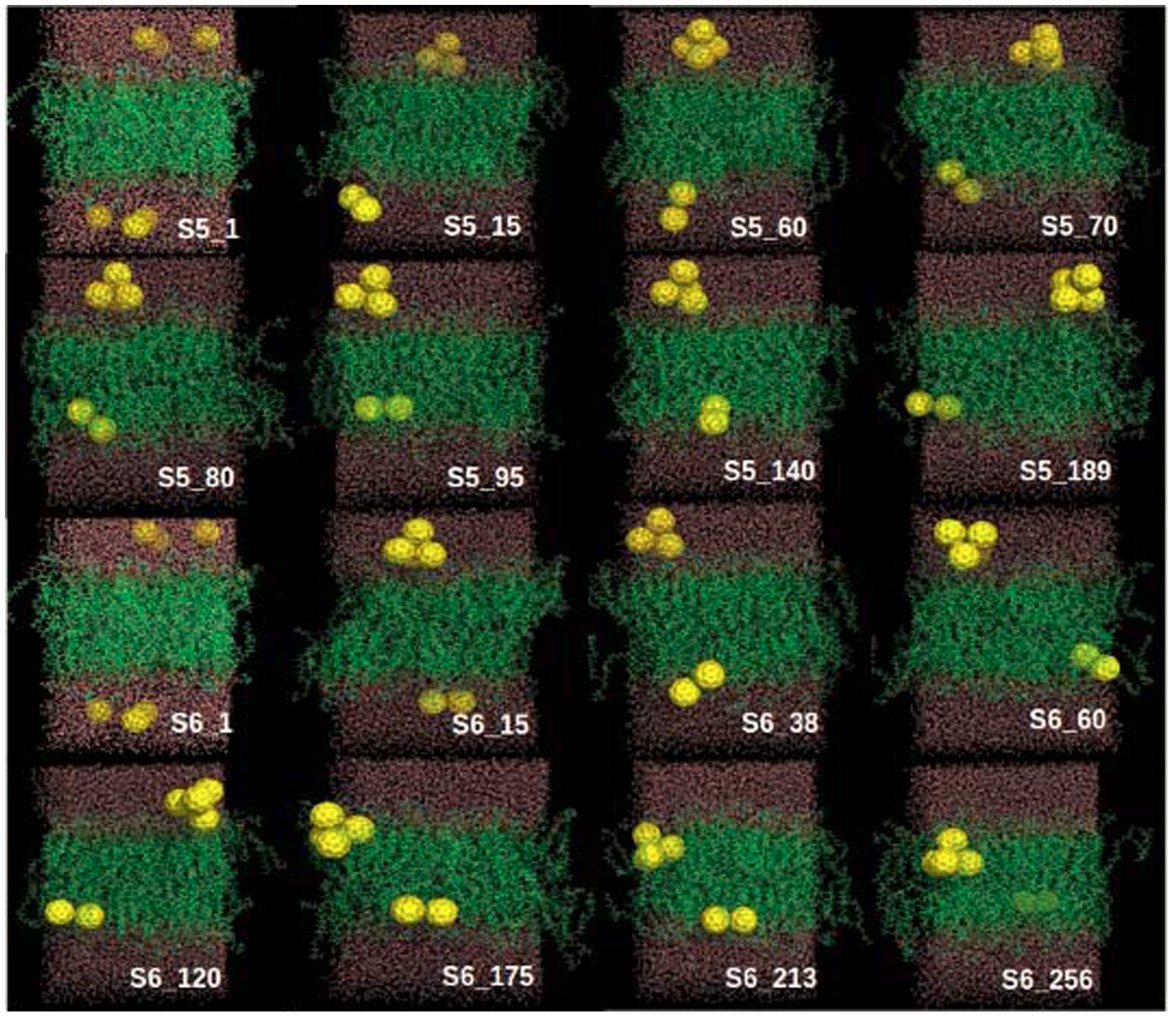
Eight representative snapshots selected according to the simulation time series (S5 and S6). The six fullerenes, DOPC lipids and water molecules are shown as yellow spheres, green lines and red lines, respectively.

In order to characterize the dynamic penetration process of fullerene nanoparticles into the DOPC membrane, some of the representative snapshots at various simulation time points are picked out and shown in Fig. 3. The representative snapshots for other simulation trajectories are shown in Fig. 4 and 5. Within short simulation time, part of the fullerene particles start to aggregate in the water phase (i.e., outside of the lipid membrane), through the driving force of hydrophobic interactions. The assembled small cluster contains 2∼3 fullerenes. Then this small cluster continues to grow by adding more free particles, and constantly adjust its structure. At this stage, the fullerene aggregates mainly locate at the interface between water and lipid membrane phases. Thereafter, the fullerene cluster begins the penetration process into the lipid membrane.

It is noticed that in one trajectory (S1) the cluster formed by all 6 fullerenes does not enter the membrane within the 200-ns simulation time (see S1 in Fig. 4). In all other simulations (Fig. 3 and 5), the clusters consisting of 4 or 5 fullerenes eventually penetrate into the bilayer. As found in previous studies, a single fullerene can easily pass through the head group region, while larger clusters need much more time to enter the membrane. [33] Lee and Chang’s pulling simulation on the permeation of one single fullerene from outside (water phase) into the DMPC membrane revealed a 15 kcal/mol energy barrier. [28] In our simulations, the fullerene cluster would possibly feel even higher energy barrier to penetrate into the DOPC membrane due to their larger size. The above theoretical consensuses are supported by the experimental measurements of UV spectrum by Takeya and coworkers. [53] It is demonstrated that fullerene partitioning state into a membrane depends on the aggregation state before entering the membrane. Large fullerene aggregates formed in an aqueous solution might not enter lipid membranes, while smaller ones would enter. [54] In addition, we do not observe the translocation of the fullerene cluster through the bilayer on the 200-ns simulation timescale. And throughout all the six simulations, the fullerene clusters inside membrane do not disaggregate into free monomers, indicating that the fullerene aggregates in our system is very stable.

From the simulations, we find two dominant penetration modes of the fullerene cluster into the lipid membrane. One mode is that the cluster assembly is finished in advance in the aqueous phase before entrance into the bilayer. The other mode is that the cluster assembly occurs simultaneously with the penetration process. Of all simulations, S3, S5, and S6 belong to the former case, while S2 and S4 have the latter mechanism. In the former mechanism, the fullerene self-assembly is almost completed before translocation into the membrane phase and the aggregate structure is essentially the same during the penetration process. In the latter case, the fullerene self-assembly is partially finished during the penetration process and the aggregate structure is accompanied by disordered lipid monomers and water molecules. The partially inserted fullerene particles string together in a line, which is enthalpically favorable for the penetration process. Eventually the fullerene aggregate is assembled into a stable irregular structure other than the patterns shown in Fig. 2.

In the S5 trajectory, the small cluster formed by two fullerenes has entered into the membrane, while the large cluster is still at the water-membrane interface. Generally the smaller the cluster the shorter time needed for the penetrating process, which indicates that the cluster size plays a role in its penetration ability. A single fullerene particle is found to insert even easily into the lipid membrane. However, neither the single fullerene particle nor cluster are found to across the lipid membrane, i.e., from the upper leaflet to the lower leaflet, within 200-ns simulations. Once the fullerene particle or cluster enters into the lipid membrane, outward translocation back to the aqueous phase is also not observed in our simulations. Notably however, there is a common feature during the insertion of the fullerene clusters into the membrane: these invading clusters tilt a certain angle and then slowly align to be parallel to the membrane plane, rather than perpendicular to the membrane plane. Such a penetrating way is supposed to bring certain structural perturbations on the lipid membrane which will be discussed later.

**Figure 6:**
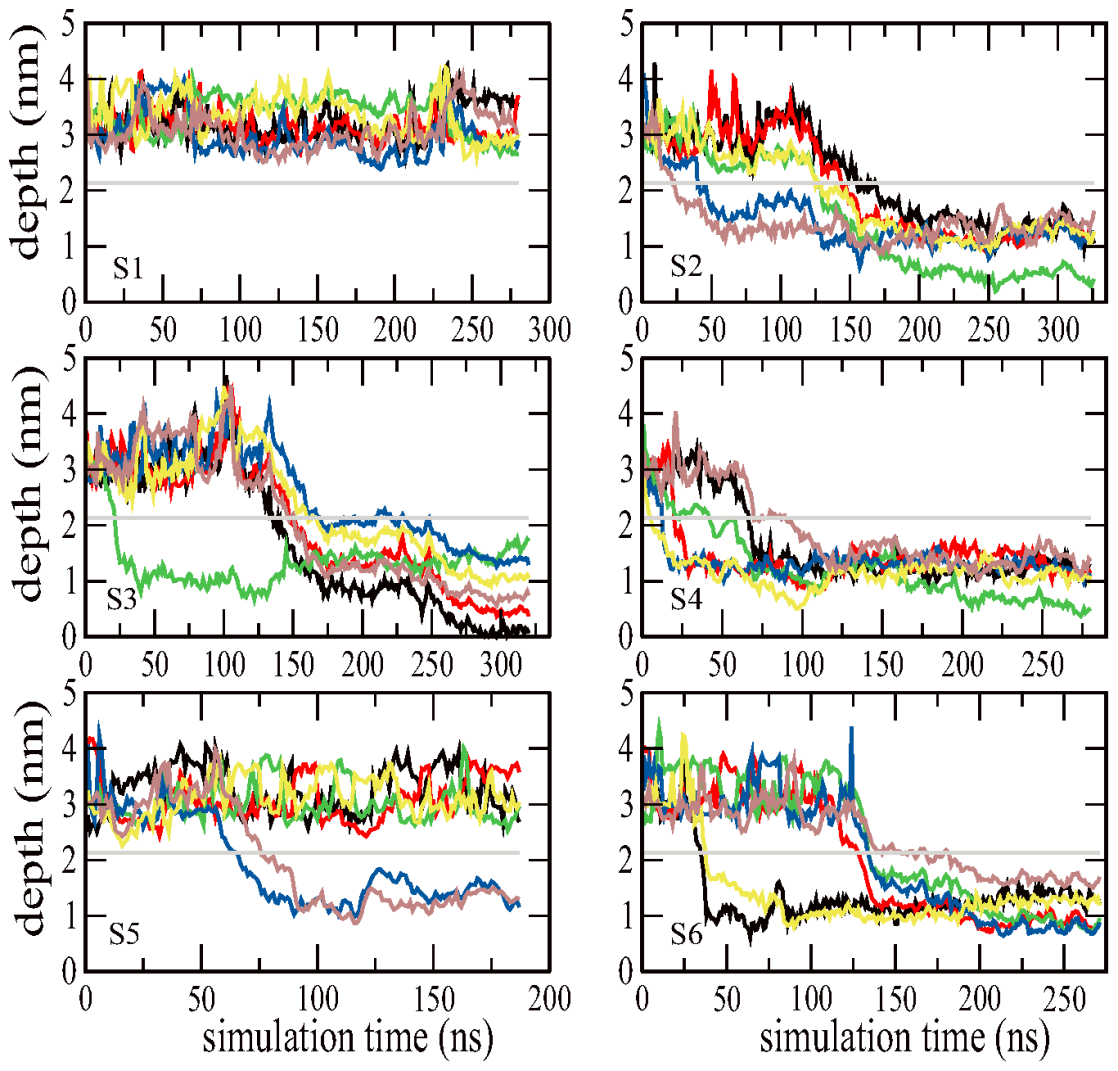
The insertion depth of each fullerene particle in the membrane as a function of the simulation time. The straight line in each subfigure is the average half thickness of the lipid membrane in the absence of fullerene, and the six colorful curves are for six fullerene particles.

In order to further quantitatively investigate the fullerene aggregation and its effect on membrane permeability, we calculated the depth of each fullerene particle inside membrane as a function of the simulation time. The straight line in each subfigure is the average half thickness of the lipid membrane in the absence of fullerene, and the zero point is the center of the membrane. Thus the straight line can be taken as the reference border of the upper/lower leaflet of bilayer membrane. The insertion depth of six fullerenes is respectively plotted in six curves as shown in Fig. 6. One can see from the subfigure S1, the six curves are all above the leaflet border within 280-ns simulations, which means the six fullerenes stay in the aqueous phase. The position of the fullerene cluster is maintained at about 1.0 nm above from the membrane surface after 80 ns. As for all other five simulations, fullerenes prefer to insert into lipid membrane, suggesting that the membrane phase is energetically favorable for fullerene cluster. In our simulation study, fullerene clusters have the preference for the region at 1.0 nm from the bilayer center, which is in agreement with previous findings. [27], [33] Some fullerene particles moves further toward the bilayer center, which implies that the fullerenes prefer to stay in the membrane center. This may be due to the lower spatial density of the membrane center, which facilitates accommodation of the fullerene invader. In simulations of S2 and S4, the distances to the bilayer center of fullerenes in the aggregation cluster are quite similar, which indicates that the particles align in a plane that is somewhat parallel to the membrane plane. As for simulations of S3 and S6, the distances to the bilayer center of fullerenes in the aggregation cluster are quite diverse, which suggests that the particles align in a plane that is somewhat oblique to the membrane plane.

**Figure 7:**
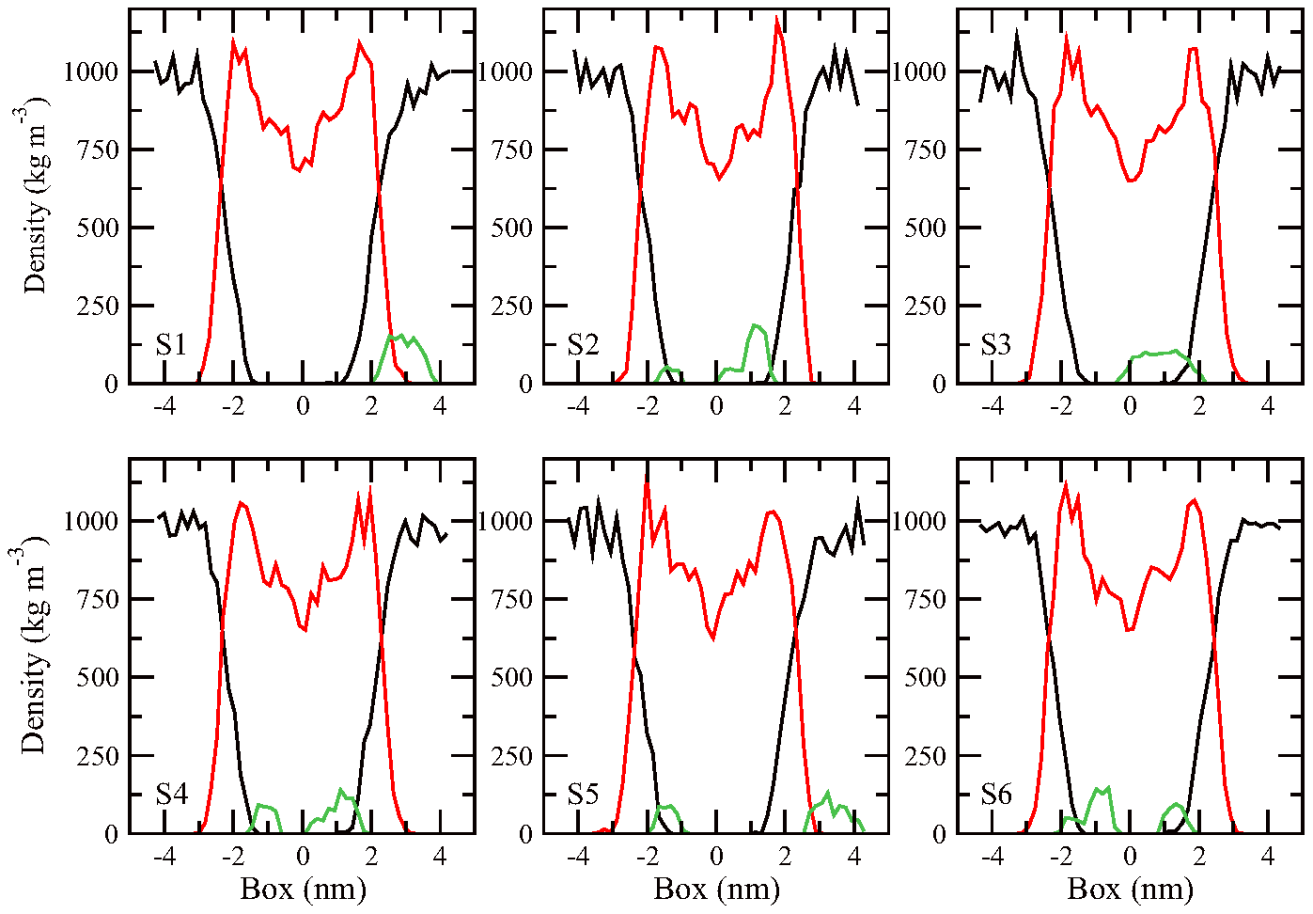
The density profiles of various components. Density profiles for fullerene (green line), the hydrocarbon tail (red line), and water molecule (black line) along the normal direction of the membrane surface.

In addition, the number density of fullerene, the hydrocarbon tail and water molecule along the direction normal to the membrane surface is calculated and shown in Fig. 7. The number density profiles show a full view of the penetration of fullerenes into lipid membrane. Except S1, fullerenes in other trajectories significantly penetrate into the membrane interior as the density profiles of lipid tail and fullerene are heavily overlapped, especially for S2, S3, S4 and S6. In addition to the fullerene insertion into the membrane, the water molecules can intrinsically also penetrate into the membrane, but mainly around the lipid head group region. The penetration ability of water molecule is in that its hydrogen atoms can form hydrogen bonds with the oxygen atoms and phosphorus atoms of the lipid head group. The insertion of water molecule with strong polarity into the hydrophobic hydrocarbon region requires high energy penalty, and thus spontaneously entering into the lipid tail region could not occur without external help. Here the penetration of fullerenes might help water molecules insert into the membrane interior. Comparing to the pure (fullerene-free) membrane system, the water molecule is more in-depth into the membrane accompanying the penetration process of fullerene cluster. From Fig. 7, it is noticed that the closest distance of water molecules to the membrane center is about 1.0 nm, while the fullerene is almost in the membrane center for simulations S2, S3 and S4. This indicates that fullerene has a higher penetration capability due to its hydrophobic nature.

### Effect of Fullerene Cluster on the DOPC Lipid Bilayer

**Figure 8:**
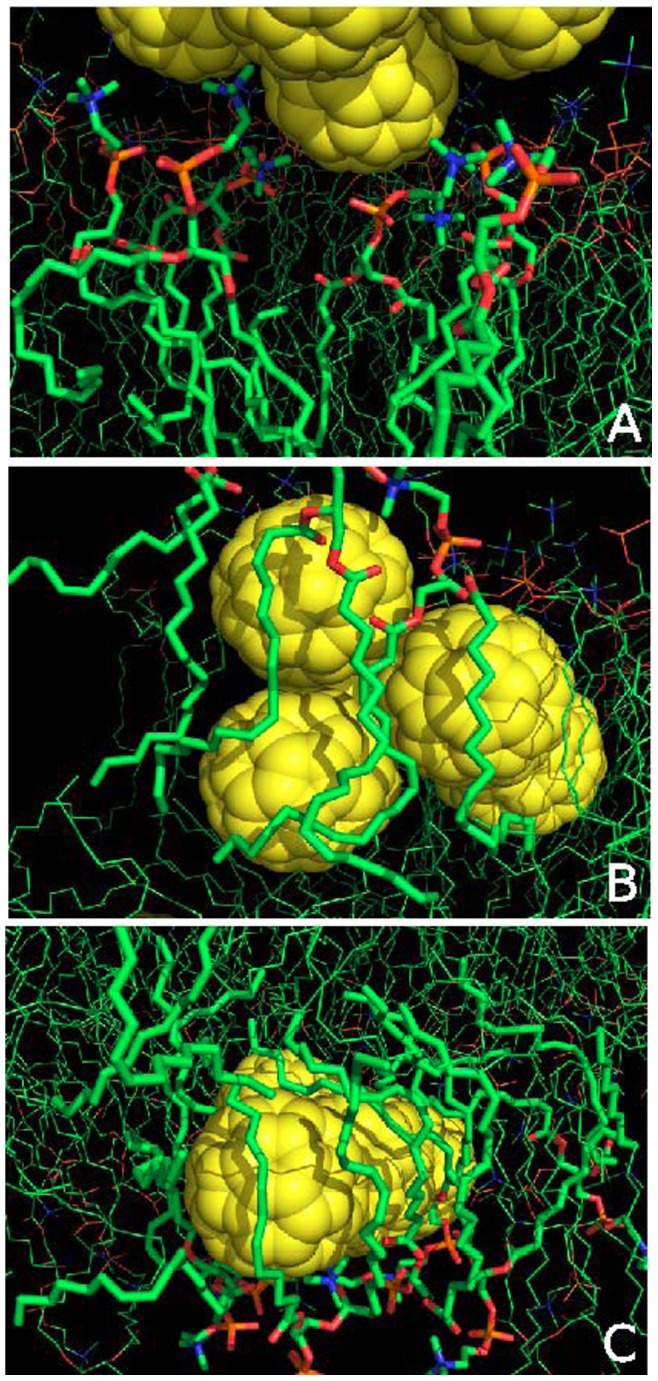
Influence of fullerene aggregate on lipid membrane. The snapshot showing the micropore in the lipid head group region induced by the fullerene cluster (a). The two snapshots demonstrating the local deformations in lipid molecules caused by the fullerene cluster (b–c).

Table 1 shows the average structural properties of DOPC lipid bilayer in the absence and presence of fullerene particles. Here the order parameter is given by [64]

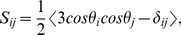
(1)where *θ_i_* is the angle between the *i*th molecular axis and the bilayer normal. Here, the molecular axis of each entire lipid is defined as the direction of its minimal moment of inertia. The order parameter is defined as S*_cd_*  =  −2/3*S_xx_*−1/3*S_yy_*. The bilayer thickness and area per lipid were calculated by using the software GridMAT-MD. [65] In comparison with the fullerene-free membrane, overally there are no significant but slight changes in the bilayer thickness, area per lipid and order parameter. In detail, the bilayer thickness is slightly increased, as a result the order parameter is a little increased. The large structural deformation is found in the simulation S2, S3, S4 and S6, where the extent of fullerene penetration is the highest as inferred from the density profile of the subfigure S2 in Fig. 5. Its bilayer thickness and area per lipid of the top leaflet are increased by up to 6% and 4%, respectively. An increase in the bilayer thickness after the insertion of fullerenes could be due to the extension of lipid bilayer as the cluster occupies certain space of the bilayer interior. The increase of the area per lipid in the top leaflet could be explained by the formation of micropore as the fullerene cluster translocation through the head groups. From Fig. 8a, we can see an obvious cavity within the head group region to accommodate the fullerene cluster penetrating into the membrane. The accumulation of fullerene nanoparticles in the membrane can lead to the widening of the membrane region, which is clearly seen in the density profile of hydrocarbon tails of DOPC lipids as shown in Fig. 7. Yang and Wang investigated two-component Langmuir monolayers of DPPC/fullerene by recording surface pressure/area and surface potential/area isotherms and by direct Brewster angle microscopy imaging. [66] It showed that the isotherms and elastic modulus versus *π* curves of the DPPC/fullerene were remarkably similar to the pure DPPC monolayers even at a molar fraction as high as *Xc*60  =  0.3, suggesting little effect of fullerene. In our simulation, the ratio of the fullerene/DOPC is about 0.05, which is smaller than the experimental ratio. In agreement with the above experimental findings, the fullerenes in our simulation system also have little effects on the structural properties of the DOPC membrane. We suppose that the reason is mainly due to the membrane fluidity that can endure the insertion of low-content of fullerene.

**Table 1:** Structural properties of DOPC lipid membrane.

	Pure	S1	S2	S3	S4	S5	S6
**Thickness** (*nm*)	4.26±0.01	4.48±0.04	4.38±0.04	4.52±0.03	4.29±0.01	4.25±0.04	4.19±0.05
**Area per lipid** (10^−1^ *nm* ^2^)	5.99±0.02	6.13±0.02	6.23±0.04	6.14±0.02	6.23±0.02	6.17±0.01	6.23±0.04
**Order parameter**	0.46±0.02	0.51±0.01	0.51±0.01	0.52±0.01	0.50±0.01	0.51±0.01	0.49±0.01

In order to explore the structural influence of fullerene aggregate on the integrality of lipid membrane, representative snapshots are displayed to show the detailed local conformations of the lipid membrane (see Fig. 8). In the snapshot from the simulation S1, the fullerene aggregate remains in the water phase but in proximity to some lipid head groups. These lipid head groups are squeezed to form a micropore to accommodate the fullerene cluster (Fig. 8A). The two snapshots in Fig. 8B and 8C are respectively chosen from the simulations S2 and S3, where the fullerene cluster has inserted into the lipid membrane. The fullerene surface is wrapped by lipid monomers due to the strong dispersion interaction. The tails of these lipid monomers are no longer stretching along the membrane normal direction, but conformationally distorted. In all, although the integrality of the whole lipid membrane is not greatly affected by fullerene cluster, its local structure is obviously disrupted.

**Figure 9:**
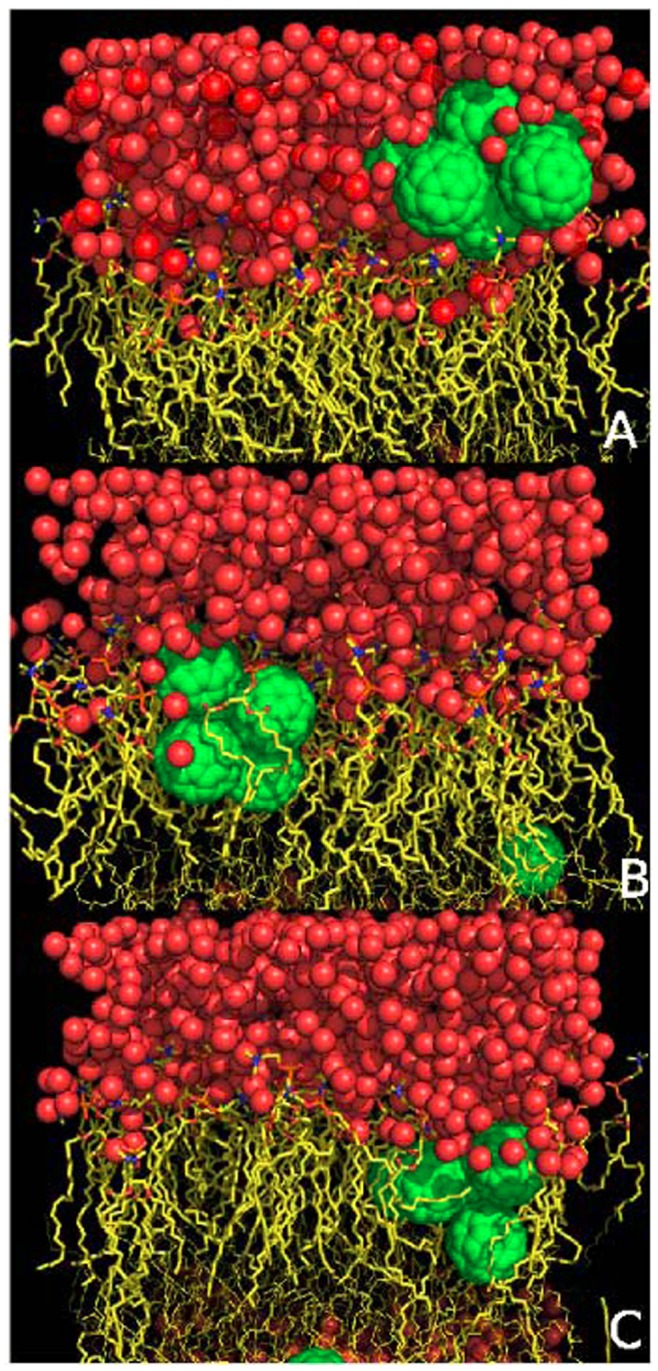
Snapshots to illustrate the penetration of water molecules into lipid membrane. 1) cluster in the head group region (the first snapshot); 2) cluster inside the membrane (the last two snapshots). The red balls are for water molecules and the green spheres are for fullerene particles.

Meanwhile, accompanying with the deformation in the local structure of the lipid membrane induced by the fullerene cluster, water molecules are found to penetrate into the membrane. Quite a few water molecules are noticed to penetrate into the lipid membrane through the micropore as illustrated in Fig. 8A (see Fig. 9A). Water molecule penetration is also found in simulations where the fullerene cluster is inserted into the membrane (Fig. 9B and 9C). This suggests that the penetration of the fullerene cluster would lead to the partial leakage of membrane. Certainly, this water leakage may also be due to the adhesion of water molecules on the fullerene surface. In all, the average structural properties of DOPC lipid bilayer are not significantly affected by the fullerene penetration. Thus, the insertion of small fullerene cluster does not exert a great mechanical impact on the integrality of the whole lipid membrane. However, the fullerene cluster results in locally conformational rearrangements of the lipid monomers, which leads to local deformation of the lipid membrane, and further partial leakage of membrane.

## Conclusions

Study on the self-assembly and translocation of carbon nanoparticles across cell membrane concerns not only the applications in nano-materials and nano-biology sciences, but also the close attention to the biosafety issue. Previously, the structure and thermodynamic stability of fullerene clusters in a non-membrane phase have been extensively investigated. [57]–[62] In the presence of membrane, both experiments and simulations confirmed that fullerenes partition to the membrane interior, however experimental information on the location of fullerene molecules was only qualitative. [54] Although some degree of aggregation was confirmed by most experiments, the extent of the aggregation was uncertain. [54] By computer simulation, single nanoparticle translocation through lipid membrane was studied by both all-atom model [25]–[28], [34], [41] and coarse-grained (CG) model. [33], [35], [37] In addition, using coarse-grained models, the rupture behavior of lipid membrane by multiple fullerene nanoparticles, [28], [33], [36] as well as the influences of hydrophobicity of nanoparticles on lipid membrane permeability have been studied. [36] However, the self-assembly of multiple fullerene nanoparticles and the resulting influence on lipid membrane was less reported. In this work, the self-assembly of fullerene nanoparticles and the resulting influence on the dioleoylphosphtidylcholine (DOPC) lipid membrane were explored by using all-atom molecular dynamics simulations with explicit solvents. Previous CG models and atomistic simulations indicated that fullerene dimerization in a lipid membrane is less favorable than in pure alkanes. Our study shows that fullerene aggregation is happened either before or accompanying the penetration process into the lipid membrane. Our simulation results confirm that gathered small fullerene cluster can invade lipid membrane, not only can the single fullerene nanoparticle. And the simulations show a vivid dynamic picture on the growth of fullerene clusters and penetration into lipid membrane, as well as the resulting influences on the membrane integrity. Our simulations show two pathways: 1) assembly process is completely finished before penetration; 2) assembly process coincides with penetration. This indicates a dynamic assembly mechanism. In addition, the diverse microscopic stacking mode (i.e., equilateral triangle, tetrahedral pentahedral, trigonal bipyramid and octahedron) of these small fullerene clusters are well characterized. Thus, the crystalline nature of the fullerene cluster found by experiments is also reflected in the simulated small fullerene cluster. Specifically, our simulations provide a detailed high-resolution characterization of the microscopic structures of the small fullerene clusters. Further, the effects of the gathered small fullerene clusters on the structure of the lipid membrane are investigated and discussed.

Notably, the lipid tails around the inserted fullerene aggregate are found wrapped on the surface of fullerene, instead of being in the normal stretching conformation (Fig. 8). Sayes *et al.* found the signs of leaky membranes and lipid oxidation after the exposure to the toxic concentrations of fullerenes. [19], [20] In our simulations we find no significant leakage, which may be due to the unreached toxic concentrations of fullerene. We only observe a few water molecules in the micropore caused by the fullerene aggregate. And the lipid membrane’s global properties (thickness, area per lipid and order parameter) are only slightly altered. From the above results, we conclude that the small fullerene cluster does not significantly cause severe mechanical damage to the lipid membrane, but only structural changes in the local region. Certainly, the simple membrane model system is not accurate enough to provide a complete picture of the cytotoxicity of nanoparticles. As an example, the invaded airborne carbonaceous nanoparticles were found to stop in the brain, which change the brain activity and affect the intracellular signaling and protein expression. [67], [68] Deeper understanding of the nanoparticle’s biological implications deserves more investigations and verifications in further both experimental and simulation researches. Nevertheless, our simulation results confirm that small fullerene cluster can invade lipid membrane. And although the penetration of small fullerene cluster does not drastically affect the global integrity of simple lipid membrane, it has significant adverse effects on the lipid structure in the local domain of DOPC membrane, which suggests the prerequisite of high-content fullerene for cytotoxicity. In summary, our simulation study reveal a microscopic picture on the self-assembly behaviors of fullerene nano-particles and resulting effect on the lipid membrane.
